# The relationship between individual sensitivity to music reward and rhythmic processing

**DOI:** 10.1007/s00426-026-02276-8

**Published:** 2026-04-09

**Authors:** Eleonora Fullone, Daniele Gatti, Giorgio Lazzari, Luca Rinaldi, Carlotta Lega, Laura Ferreri

**Affiliations:** 1 Department of Brain and Behavioral Sciences, University of Pavia, Pavia, Italy; 2 School for Advanced Studies - IUSS, Pavia, Italy; 3 Department of Medicine and Surgery, University of Parma, Parma, Italy; 4 Applied Psychology Unit, IRCCS Mondino Foundation, Pavia, Italy

**Keywords:** Rhythm, Rhythmic processing, Musical hedonia, Music reward, Individual differences

## Abstract

Rhythm is a fundamental aspect of music, naturally developing in humans, engaging perceptual, emotional, and motor processes, and in turn relying on multiple cognitive functions such as memory and attention. Rhythm is also closely linked to music reward, as musical pleasure arises from making and resolving predictions about temporal structures. However, this relationship remains largely unexplored. To fill this gap, in the present study, a sample of non-musicians (i.e., with less than 3 years of formal musical training, *N* = 121) performed tasks and questionnaires assessing the multidimensional nature of rhythmic abilities and the individual sensitivity to music reward (i.e., musical hedonia). Rhythmic skills were indexed measuring rhythm production (Finger-Tapping), perception (CA-BAT), and memory (MET), while musical hedonia was evaluated using the extended version of the Barcelona Music Reward Questionnaire (eBMRQ). Results indicated that individuals with higher musical hedonia showed higher timing consistency in the Finger-Tapping task and increased sensitivity to asynchrony variations in the CA-BAT. Additional analyses restricted to participants with 0 years of formal musical training showed a similar effect for the MET task, with higher musical hedonia predicting higher accuracy. Beyond this general trend, we also found specific subcomponents of the eBMRQ to be specifically related to enhanced rhythmic perception, production, or memory outcomes. These findings clearly demonstrate a relationship between rhythmic skills and individual sensitivity to music reward, suggesting that musical hedonia is strongly associated with rhythmic processing.

**Supplementary Information:**

The online version contains supplementary material available at 10.1007/s00426-026-02276-8.

## Introduction

Regular patterns are fundamental to both natural and human-made systems, providing structure, predictability, and coherence. In soundscapes, these patterns are organized temporally, with music and its rhythmic elements serving as a prime example of this organization.

Rhythm is defined as the patterns and organization of durations and inter-onset intervals of sounds and silences over time (Fiveash et al., 2023). As humans, we can recognize patterns in the lengths of notes and pauses in music, often leading us to synchronize movements like clapping to the beat. This ability arises from our perception of the pulse or beat and the underlying temporal structure, known as meter (Levitin et al., 2018). Once the beat and meter are identified, rhythmic information and expectations of temporal regularity align with the listener’s internal state, enabling bodily movements to synchronize with the music. On a neural level, neural entrainment (neurons resonating at the beat frequency; Large, 2008; Nozaradan et al., 2011) and sensorimotor integration processes activate both auditory and motor brain regions, including the premotor cortex, supplementary motor area, cerebellum, and basal ganglia (Chen et al., 2008; Lega et al., 2016; Rauschecker, 2011).

The ability to find the beat and the spontaneous inclination to synchronize movement with it emerge early in infancy (Winkler et al., 2009) and develop even in the absence of formal musical training (Leow & Grahn, 2014; Repp, 2010). However, substantial individual differences exist among healthy adults in their capacity to perceive and produce rhythm (Grahn & Schuit, 2012; Mills et al., 2015; Thompson et al., 2015). These variations are typically assessed through test instruments designed to capture the complex, multi-faceted nature of rhythm processing, and are influenced by both musical experience (Grahn & Rowe, 2009; Nave-Blodgett et al., 2021) and genetic factors (Niarchou et al., 2022). Fiveash et al. (2022) explored the multidimensionality of rhythmic abilities in the general population and identified three main dimensions that correspond to distinct yet interrelated components: rhythm production, beat-based rhythm perception, and sequence memory-based rhythm processing.

Rhythm also plays a key role in music-induced emotional responses. The rhythmic properties of music can affect subjective ratings of valence, arousal, and enjoyment by modulating heart rate and respiration (Juslin, 2013; Koelsch & Jäncke, 2015; Trost & Vuilleumier, 2013). In this context, it has been proposed that the brain’s ability to predict the timing of music, based on beat and meter, is essential for enhancing enjoyment through sensorimotor synchronization. Groove is usually defined as the pleasurable desire to move to music. Research in music cognition suggests that listeners’ expectations about rhythm influence two key aspects of musical experience: the urge to move in sync with the music and the level of enjoyment (e.g., Janata et al., 2012; Koelsch et al., 2019; Spiech et al., 2022, 2024). Specifically, rhythms of medium complexity, striking an optimal balance between confirming and violating expectations over time, elicit stronger groove sensations compared to rhythms of lower and higher complexity (Matthews et al., 2019). At the neural level, experiencing groove engages not only the motor network but also the brain’s mesolimbic reward pathway (Matthews et al., 2020), which is directly linked to musical pleasure (Blood & Zatorre, 2001; Ferreri et al., 2019; Salimpoor et al., 2011). As a result, rhythmic engagement through listening and movement can trigger a variety of emotional responses, encompassing both affect and pleasure. These findings underscore the strong link between rhythm and reward, raising new questions about how they may interact. While synchronizing to rhythm might create an emotionally rewarding experience through predictive processes, the degree to which music reward is tied to rhythm processing remains unexplored (Fiveash et al., 2023).

Importantly, individuals differ substantially in their sensitivity to music reward, also known as musical hedonia. This is generally assessed by the Barcelona Music Reward Questionnaire (BMRQ, Mas-Herrero et al., 2014) and its extended version, eBMRQ (Cardona et al., 2022), which measures individuals’ musical hedonia through different subscales (Musical Seeking, Mood Regulation, Emotion Evocation, Sensory-Motor, Social Reward, Musical Absorption). Higher scores on this questionnaire are associated with higher pleasurable responses during music listening and increased activation of the dopaminergic reward system (Martínez-Molina et al., 2019). In contrast, individuals with low musical hedonia report low to no pleasure in response to music and show decreased connectivity between the auditory cortex and the reward system (Belfi & Loui, 2020; Martínez-Molina et al., 2016).

Individual differences in rhythm and music reward processing may therefore provide a valuable perspective for exploring the connection between these two domains. One hypothesis is that there may be a link between individuals’ musical hedonia and their rhythmic processing abilities, suggesting that individual ability to derive pleasure from music might influence rhythmic processing. To investigate this, we conducted a study that assessed rhythmic abilities in the general population using three distinct tasks indexing rhythm production, perception, and memory, and evaluated musical hedonia through the eBMRQ questionnaire.

## Materials & methods

### Participants

One hundred and twenty-one healthy adult volunteers (40 m, 81f), aged between 18 and 35 years (Mean age = 25.79 years, *SD* = 3.95 years), non-musicians took part in the present study. In the recruitment procedure, we defined non-musicians as individuals with less than 3 years of formal musical training (Bidelman et al., 2013; Mankel & Bidelman, 2018). To further assess potential musicianship, we also employed two subscales of the Goldsmiths Musical Sophistication Index, namely, the Perceptual Abilities and Musical Training subscales (Müllensiefen et al., 2014; see Table 1 for detailed descriptives). The sample size was estimated a-priori, following the common practice of enrolling at least one hundred participants in studies investigating individual differences (see e.g., Brysbaert, 2019). Participants were recruited via announcements on the University Department’s website. Due to technical issues that occurred during data acquisition, two participants were not included in two of the three experimental tasks. All participants gave written informed consent to the study in accordance with the procedure approved by the local Ethics Committee (Department of Brain and Behavioral Sciences, University of Pavia).

**Table 1:** Descriptive statistics for participants’ age and scores on the Gold-MSI Musical Training and Perceptual Abilities subscales. “Percentile Gold-MSI” values refer to the participants’ median scores on each Gold-MSI subscale relative to the UK normative sample reported in Müllensiefen et al., 2014 (File S1, Table S3)

Variable	Mean	Median	SD	Min	Max	*n*	Percentile (Median) Gold-MSI
Age (years)	25.79	25	3.95	19	35	121	–
Musical Training (Gold-MSI subscale)	18.05	16	8.03	7	44	121	~ 23-24th
Perceptual Abilities (Gold-MSI subscale)	43.99	43	7.09	25	63	121	~ 15-18th

### Measures

**Fig. 1:**
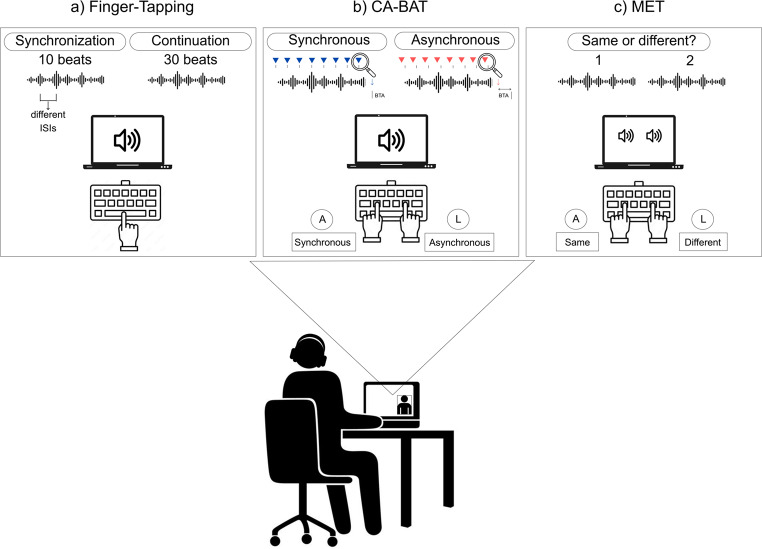
Illustration of the experimental tasks adopted to test **(a)** rhythmic production (Finger-Tapping), **(b)** rhythmic perception (CA-BAT), **(c)** rhythmic memory (MET)

We assessed participants’ musical hedonia through the eBMRQ, and their rhythmic abilities through three tasks in the fixed order summarized in Fig. 1.

#### Extended barcelona music reward questionnaire (eBMRQ)

The Italian version of the *eBMRQ* (Carraturo et al., 2023) was used to assess individual differences in musical hedonia. Participants rated their agreement to 24 questions using a 5-point scale (from 1, completely disagree, to 5, completely agree). These items are divided into six sub-scales: (1) Musical Seeking (i.e., seeking formal music knowledge); (2) Mood Regulation (i.e., music as a mood or hedonic regulator); (3) Emotion Evocation (i.e., elicitation of emotional responses to music); (4) Sensory-Motor (i.e., spontaneous body synchronization and coordination to music); (5) Social Reward (i.e., music as a contributor to social bonds); and (6) Musical Absorption (i.e., music-driven transcendence experience). The maximum possible score of the eBMRQ, given by the sum of the scores obtained in each subscale, is 120, and the minimum is 24.

#### Finger-tapping task

The Finger-Tapping was used to assess the production rhythmic ability. The task is based on a sensorimotor synchronization in which an action (i.e., finger tapping) is temporally concurrent with a predictable external stimulus (i.e., a sound at 441 Hz presented via headphones). The task consisted of two consecutive phases: Synchronization and Continuation (Braun Janzen et al., 2014; Michon, 1967; Stevens, 1886). For each trial, participants first synchronized their movements by pressing the spacebar on the keyboard with an isochronous auditory stimulus for 10 taps. After the stimulus stopped, participants were instructed to continue tapping at the same rate for 30 additional taps (i.e., Continuation phase). The stimulus was presented at different tempi (inter-stimulus interval, ISI) in a randomized order: 900 ms, 975 ms, 1005 ms, 1125 ms, 1200 ms, 1275 ms. Since we were interested in individual differences, the decision to use near-second ISI was based on the fact that non-musicians tend to show greater variability with a slow beat (from 1000 to 3500 ms; Repp & Doggett, 2007). Each ISI was presented 2 times, for a total of 12 trials, randomly presented. Participants were familiarized with the task via presentation of a single practice trial. They were instructed not to use any mental strategy (e.g., counting). No feedback was provided during the task.

#### Computerized adaptive beat alignment test (CA-BAT)

An adapted version of the CA-BAT (Harrison & Müllensiefen, 2018) was used to assess individual beat perception ability, which involves the process of predicting and extracting the beat or pulse from an external musical stimulus (Patel & Iversen, 2014). Participants listened to 17 musical tracks, each lasting around five seconds, accompanied by a metronome-like beep track. For each musical track, they had to determine whether the beep track was aligned (synchronous) or misaligned (asynchronous) with it. This adaptation of the original task, which required identifying the synchronous track between two sequentially presented options, was modified to eliminate the comparative judgment aspect present in the memory task. The asynchronous condition was manipulated by introducing varying degrees of misalignment. The extent of misalignment was quantified using beat-track accuracy (BTA), an index ranging from 0 to 1 (Harrison & Müllensiefen, 2018). Smaller BTA values indicate a larger displacement, while the maximum value of 1 corresponds to perfect overlap between the metronome and the beat. For the current experiment, six BTAs (0.50, 0.60, 0.70, 0.80, 0.90, and 1.0) were used, resulting in a total of 102 trials (17 tracks × 6 degrees of misalignment) presented randomly. Participants were familiarized with the task through two (asynchronous and synchronous) practice trials, and provided their answers using keyboard buttons. No feedback was given during the task.

#### Musical ear test (MET)

The MET (Wallentin et al., 2014; online version, Correia et al., 2021) was used to evaluate music memory abilities. The original version includes two subtests, Melody and Rhythm, each with 52 trials. Since we were specifically interested in the rhythmic component, only the Rhythm Memory task was used. In each trial, participants were exposed to two brief rhythmic musical segments: an initial target track followed by a comparison track. Participants had to indicate whether the comparison track was rhythmically identical to the target track. Half of the trials presented identical rhythmic tracks, and the other half differed. All musical segments shared the same metrical structure (4/4 time), with the beat signaled by a lower-amplitude metronome sound, and tempo (100 beats per minute), and they were presented in a random order. Participants familiarized with the task via the presentation of two (identical and different sequences) practice trials, and provided their answers through keyboard buttons. No feedback was provided during the task.

### Procedure

The experimental procedure took place online. Participants first completed the informed consent and online questionnaires: demographic data, musical expertise assessment (see Participants section), and the eBMRQ. Then, their rhythmic processing was tested. To ensure a large sample with high data quality, a hybrid testing approach was used for this phase. The experimenter connected with each participant individually via Zoom to provide instructions, resolve technical issues, and ensure proper task performance (e.g., focused attention, quiet environment, correct headphone usage). Participants completed the three rhythmic tasks: Finger-Tapping (Fig. 1a), CA-BAT (Fig. 1b), and MET (Fig. 1c). The experimental session lasted around 30 min. The questionnaires were administered through Google Forms. The rhythmic tasks were programmed using PsychoPy (Peirce, 2007) and delivered via the Pavlovia platform (Open Science Tools, Nottingham, UK).

### Data analysis

Analyses were performed using R-Studio (RStudio Team, 2015). Data were analyzed using linear models for simpler relationships (MET task) or mixed-effects models (Finger-Tapping and CA-BAT tasks), which include both fixed and random effects (e.g., for participants and items) to account for non-independence at both levels (Baayen et al., 2008). Linear mixed models (LMMs) and generalized linear mixed models (GLMMs) were implemented with the lme4 R package (Bates et al., 2015), with GLMMs estimated on a binomial distribution. Data from each rhythmic task were analyzed separately.

#### Finger-tapping task

Here, we examined how participants’ rhythm production ability, assessed during the Continuation phase of the Finger-Tapping task, is differentially associated with individual differences in sensitivity to music reward, as measured by the eBMRQ. To do this, the dependent variable was the difference (i.e., the delta) between each response’s timing and the ISI of a given trial during the Continuation phase (deltaRTs). This measure reflects the temporal proximity of each tap to the target rhythm: the closer the value is to zero, the higher the accuracy. Negative values indicate a tap occurring before the expected timing, whereas positive values indicate a tap occurring after the expected timing. DeltaRTs exceeding 2000 ms were removed from the analysis (12% of the trials). To examine the relationship between participants’ rhythmic abilities and musical hedonia, we estimated an LMM with deltaRTs as the dependent variable. The model included the order of taps within each trial in the Continuation phase (i.e., the 30 consecutive taps; or order of taps), participants’ eBMRQ values, and their interaction as continuous fixed factors. Participants and ISI were included as random intercepts. The analysis of the Continuation phase in this paper provides insight into participants’ ability to maintain tapping consistency over time, extending beyond mere performance accuracy (e.g., Perrone et al., 2023).

#### CA-BAT task

The aim was to investigate how individual differences in sensitivity to music reward relate to participants’ rhythmic perception in the Computerized Adaptive Beat Alignment Task (CA-BAT). Thus, the dependent variable was the proportion of asynchronous responses. Trials with response latencies longer than 10,000 ms or shorter than 1,000 ms were excluded from the analysis (2.3% of trials). As a first sanity check, we estimated a GLMM with participants’ proportion of asynchronous responses as the dependent variable and BTAs as a continuous fixed factor (1.0 as the synchronous BTA, and 0.50, 0.60, 0.70, 0.80, 0.90 as the asynchronous alternatives). Participants and items were included as random intercepts. Then, to investigate the role of musical hedonia on participants’ rhythmic perception, we estimated a GLMM with participants’ proportion of asynchronous responses as the dependent variable. The model included BTAs and participants’ eBMRQ values as continuous fixed factors, along with their interaction. Participants and tracks were included as random intercepts.

#### MET task

To examine how individual differences in sensitivity to music reward relate to participants’ performance on the Musical Ear Test (MET), we computed the d’ as a reliable, bias-free estimate of sensitivity in recognition memory tasks (see e.g. Ferreri & Rodriguez-Fornells, 2017). More specifically, the dependent variable was participants’ d’, calculated as the difference between the z-scores of hit rates (tracks identified as identical when they were the same) and false alarm rates (tracks identified as identical when they were different). To investigate the role of musical reward in task performance, we estimated an LM with participants’ d’ as the dependent variable and eBMRQ values as the continuous factor.

#### Additional analyses on the role of musicianship

To complement the main analysis, we further explored potential effects of musical training by investigating the subgroup of participants with 0 years of formal musical training. To this end, we conducted an additional analysis using only participants with no formal musical training at all (*N* = 54), as assessed with the first question of the Musical Training subscale of the Gold-MSI questionnaire (i.e., “I engaged in regular, daily practice of a musical instrument (including voice) for 0 years”). Thus, we applied the same approach described in the main analyses.

We also conducted further exploratory analyses investigating whether individual differences in perceptual abilities (assessed with the Gold-MSI Perceptual Abilities subscale) and their interaction with musical hedonia (eBMRQ) influence performance across the Finger-Tapping, CA-BAT, and MET tasks (details reported in the Online Resource).

#### eBMRQ subscales and rhythmic processing

As a further analysis, we explored which subscale of the eBMRQ questionnaire best explained participants’ responses in each of the three tasks. In the absence of a significant relationship between the eBMRQ and the dependent variables, we investigated whether any subscale better indexed the observed effects. To do this, we adopted a data-driven approach. For each task, we first estimated a baseline model that included only the primary task variables (i.e., the BTA in the CA-BAT, the Continuation phase in the Finger-Tapping, or the null model in the MET). Then, for each subscale, we estimated a different model by adding it to the baseline model (in interaction in the case of CA-BAT and Finger-Tapping), allowing us to test whether the inclusion of a specific subscale improved the model fit. Thus, for each task, we ended up with seven models: one baseline model and six additional models, each incorporating one subscale. To perform the model comparison, we computed the Akaike Information Criterion (AIC) for baseline and subscale models, which measures the quality of fit of a given model to a given set of data (the lower, the better; Akaike, 1998). For each task, we subtracted the AIC of the baseline model from the AICs of the other models. Negative values can be considered as indicative of evidence in favor of the full model, with ΔAIC < -2 indicating substantial support in favor of the full model (Hilbe, 2011).

For the Finger-Tapping, we used mixed-effects linear regression models to examine the effect of different eBMRQ subscale predictors on the variable delta, with the Continuation phase as a primary predictor and random intercepts for participants and ISI. The baseline model included only the Continuation phase (i.e., order of taps) as a fixed effect, while the other six models each included the interaction between the Continuation phase and a specific musical subscale. For the CA-BAT, we used a similar approach with mixed-effects logistic regression models to examine the influence of different musical traits on participants’ proportion of asynchronous responses while accounting for random intercepts for participants and tracks. The baseline model included only the fixed effect of stimuli, with each of the six subsequent models adding an interaction term between BTAs and one of the subscale predictors. For the MET, a set of linear regression models was estimated to investigate the effect of the various subscale predictors on the outcome variable d’. The baseline model was the null model in this case, while each subsequent model added a single subscale as a continuous predictor.

## Results

### Finger-tapping task

**Fig. 2:**
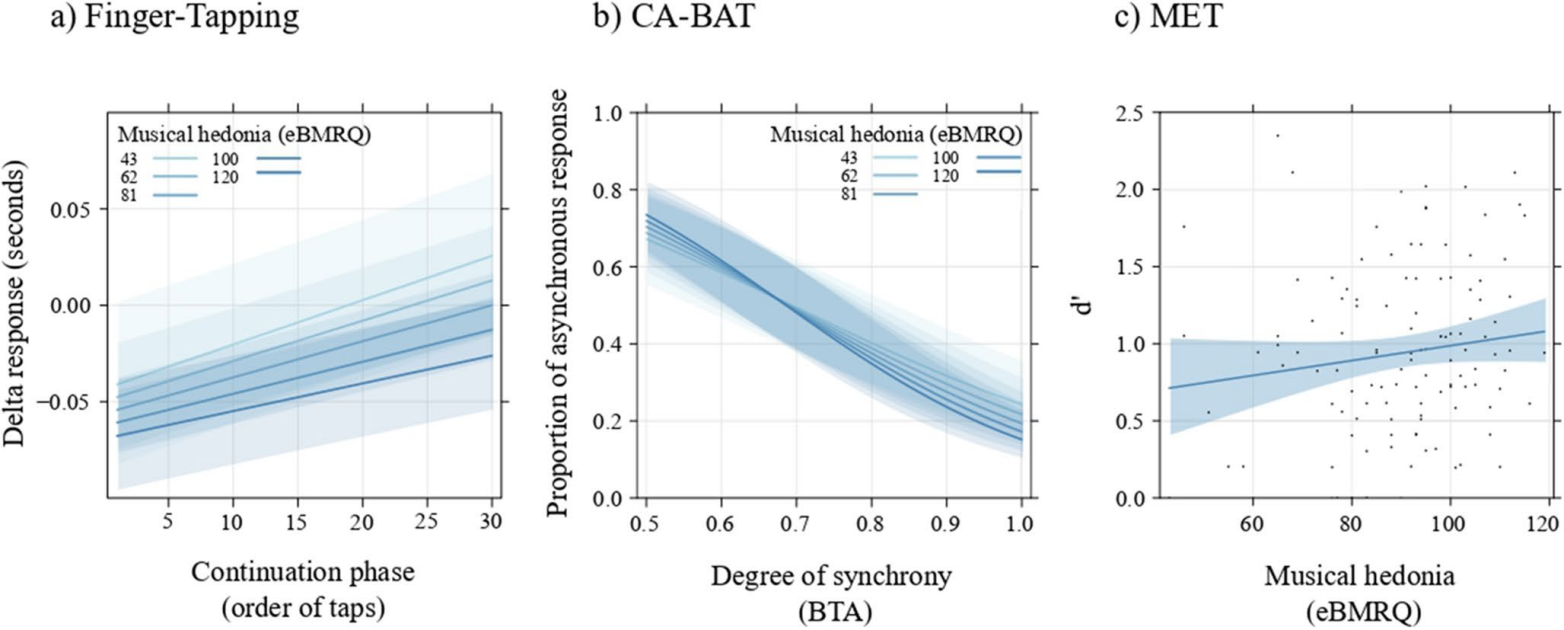
Plots illustrating the main findings of the three rhythmic processing tasks. **a)** Results of the model estimated on Finger-Tapping data, showing the influence of musical hedonia (eBMRQ) on the relationship between the independent variable, the Continuation phase (order of taps represented), and the dependent variable, the delta between the response timing of each response and the ISI. The higher the musical hedonia, the higher the time consistency (the lower the participants’ tendency to have longer responses). **b)** Participants’ slope and relationship between BTAs and eBMRQ values in predicting participants’ behavior in the CA-BAT task. The slope shows that the probability of answering that the track is asynchronous (i.e., dependent variable) decreases when the BTA (i.e., independent variable) is higher (where 1 is the synchronous track). The interaction with musical hedonia (eBMRQ) indicates that participants with higher eBMRQ values are more sensitive to the task. **c)** Results of the model estimated on MET task data: non-significant relationship between eBMRQ values (i.e., independent variable) and task performance (i.e., d’, dependent variable)

 The Type III Analysis of Variance using Satterthwaite’s method revealed that the main effect of eBMRQ values was not statistically significant (F(1,128.3) = 0.62, p = .434). In contrast, the main effect of the Continuation phase was significant (F(1,43381.0) = 32.97, p < .001). Additionally, the interaction between eBMRQ and the Continuation phase was also significant (F(1,43381.0) = 4.51, p = .034). As shown in Fig. 2a, these results indicate that overall participants tended to show longer responses as the trials continued (i.e., they were less accurate in Finger-Tapping over time). However, the higher the individual sensitivity to music reward (i.e., musical hedonia), the lower this tendency and thus the higher the timing consistency during each trial.

### CA-BAT task

Results showed that BTA predicted participants’ performance (χ²(1) = 375.26, p < .001), indicating that the higher the misalignment (i.e., the lower the BTA), the higher the probability of answering that the trace is asynchronous. This result is in line with the construction of the task: since higher BTAs are closer to the synchronous track, they are more difficult to individualize as asynchronous ones, while lower BTAs—being further from the synchronous track—are easier to be recognized as asynchronous. The GLMM model assessing the role of musical hedonia on the performance with the Analysis of Deviance using Type II Wald chi-square tests revealed a significant main effect of BTA (χ²(1) = 1300.55, p < .001) and a significant interaction between BTA and eBMRQ (χ²(1) = 8.34, p = .004). The main effect of eBMRQ, however, was not statistically significant (χ²(1) = 0.43, p = .512). As shown in Fig. 2b, results indicate that participants with higher musical hedonia are more sensitive to misalignment. That is, higher eBMRQ scores predicted individual sensitivity in perceiving the variations of asynchronies in the stimuli, with highly hedonic participants showing a greater tendency to respond ‘asynchronous’ for misaligned stimuli and ‘synchronous’ for aligned or nearly aligned stimuli. This, therefore, does not simply indicate better performance, but rather a heightened sensitivity to misalignment, as shown by a steeper regression line. The effect of musical hedonia decreased when approaching more synchronous musical excerpts.

### MET task

The model had an adjusted R^2^ = .01 and revealed an F(1,117) = 2.32, with a non-significant effect of individual differences in musical hedonia (i.e., eBMRQ values) on d’ (t = 1.52, b = 0.08, p = .13) (Fig. 2c).

### Additional analyses on the role of musicianship

For the Finger-Tapping task, and in line with the analysis on the whole sample of participants, the Type III Analysis of Variance using Satterthwaite’s method revealed that the main effect of eBMRQ values was not statistically significant (F(1, 55.76) = 0.20, p = 0.656). In contrast, the main effect of the Continuation phase was significant (F(1, 19350.01) = 16.57, p < .001) as was the interaction between eBMRQ and the Continuation phase (F(1, 19350.00) = 4.03, p = 0.045), confirming that, even in the subgroup of participants with 0 years of formal musical training, higher musical hedonia was associated with a higher timing consistency during each trial. Similarly, for the CA-BAT task, the GLMM model assessing the role of musical hedonia on the performance, with Analysis of Deviance using Type II Wald chi-square tests, revealed a significant main effect of BTA (χ²(1) = 445.19, p < .001), no main effect of eBMRQ (χ²(1) = 0.04, p = .844), and a significant interaction between BTA and eBMRQ (χ²(1) = 3.95, p = .047), confirming higher eBMRQ scores predicted individual sensitivity in perceiving the variations of asynchronies in the stimuli in participants with 0 years of formal music training. For the MET task, the model showed an adjusted R² = .09 and a significant overall effect (F(1,51) = 6.19). Unlike the analyses conducted on the whole sample, there was a significant positive effect of musical hedonia (i.e., eBMRQ values) on d’ (t = 2.49, b = 0.18, *p* = .02): individuals with higher sensitivity to music reward performed better in recognizing rhythmic sequences in the memory task.

In addition, exploratory analyses of the Gold-MSI Perceptual Abilities subscale revealed a significant interaction between musical hedonia (eBMRQ) and perceptual abilities for the Finger-Tapping and CA-BAT tasks, but not MET, indicating that the main effects of musical hedonia on the production and perception tasks were strongest among participants with higher perceptual abilities (see Online Resource for more detailed information).

### eBMRQ subscales and rhythmic processing

**Fig. 3:**
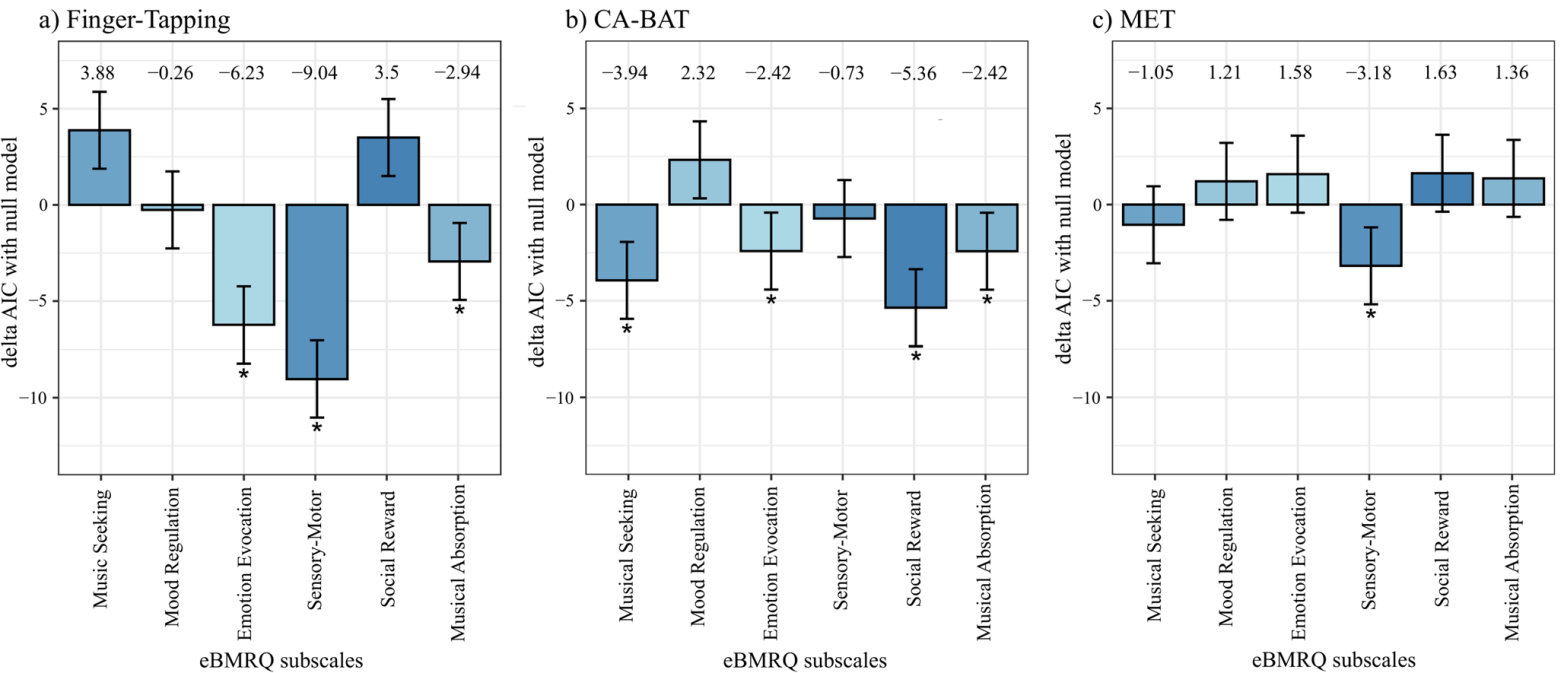
Plots illustrating the histograms of the model selection analyses for each subscale of the eBMRQ questionnaire, with ΔAIC < -2 indicating substantial support in favor of the full model. **a)** Finger-Tapping task: effects of eBMRQ subscale predictors on the variable delta, with Emotion Evocation, Sensory-Motor, and Musical Absorption subscales influencing task performance. **b)** CA-BAT task: effects of the Music Seeking, Emotion Evocation, Social Reward, and Musical Absorption subscales on participants’ proportion of asynchronous responses. **c)** MET task: effect of the eBMRQ subscale predictors on the outcome variable d’. Here, the Sensory-Motor subscale was found to be more informative than the variable indexed by the total eBMRQ

Concerning the subscales of the eBMRQ, histograms of the model selection procedures are reported in Fig. 3. Negative values lower than 2 ΔAIC points indicate that the specific model(s) outperform the baseline one. For the Finger-Tapping task, Emotion Evocation, Sensory-Motor, and Musical Absorption subscales stand out with the lowest delta AIC values, indicating their significant influence on task performance. In particular, the model including the Sensory-Motor subscale appears as the best one, with a ΔAIC= -9.04 (Fig. 3a). For the CA-BAT task, all subscales—except Mood Regulation and Sensory-Motor—exhibit ΔAIC < -2, suggesting that models incorporating interactions between stimuli and each subscale improve fit relative to the null model. Within this context, the model with the Social Reward subscale is the best-fitting one (Fig. 3b). Concerning the MET Task, interestingly, only the model with the Sensory-Motor subscale outperforms the null one with a ΔAIC= -3.18, thus indicating that this specific component is more informative than the whole variable indexed by the eBMRQ (Fig. 3c).

## Discussion

This study aimed to investigate the relationship between musical hedonia and rhythmic abilities in a general population of non-musicians. We hypothesized an association between individual sensitivity to music reward and rhythmic abilities, expecting that eBMRQ scores would reflect differences in rhythmic processing. Our findings reveal an effect on rhythm production and beat-based perception, but not memory-based rhythm processing. In the Finger-Tapping task, participants with higher musical hedonia showed greater temporal consistency (reduced delay across trials). In the CA-BAT task, highly hedonic participants were more sensitive to misalignment, as indicated by a higher probability of responding “asynchronous” when misaligned and “synchronous” when aligned or nearly aligned with the auditory sequence (see also Lazzari et al., 2025). Further analysis on participants with 0 years of formal musical training confirmed the role of musical hedonia in production (Finger-Tapping) and perception (CA-BAT) tasks, and additionally revealed its effect in the memory task (MET), showing that the relationship between individual sensitivity to music reward and rhythmic processing persists, and is even stronger considering no musical training at all. Moreover, exploratory analysis of the eBMRQ subscales revealed that distinct components may drive the association between musical hedonia and rhythmic processing. Although further analyses are needed to understand how and why the different components of music reward are associated with rhythmic skills, these findings highlight the strong link between rhythm and reward. In particular, they show that individual differences in music-induced pleasure are closely tied to rhythmic processing, particularly production and perception. Finally, supplementary exploratory analyses indicated that the effect of musical hedonia on Finger-Tapping and CA-BAT performance was strongest in participants with higher perceptual abilities (PA subscale of the Gold-MSI), whereas no such effect was observed in the MET task (see Online Resource). Although this observation is based on a single self-reported subscale, it suggests that individuals with greater musical skills may be better able to translate their enjoyment of music into enhanced rhythmic processing. This finding highlights the relevance of individual differences in understanding the relationship between music reward and rhythm, and calls for future research employing multimodal and objective assessments of musical abilities.

Rhythm, a core element of music, organizes auditory input and shapes musical emotions. Evidence shows rhythm engages perceptual and motor processes, relying on cognitive functions like time perception, working memory, and attention (Fiveash et al., 2022; Zatorre et al., 2007), which in turn influence affective responses to music (Vuilleumier & Trost, 2015; see also Cui et al., 2023; Matthews et al., 2024). On a neural level, motor areas like the premotor cortex and basal ganglia are activated during both motor synchronization and rhythm perception, explaining the natural urge to move with music, known as groove. This effect arises from hierarchical predictive processes that help listeners anticipate rhythmic patterns based on prior musical context (Vuust & Witek, 2014). This anticipation is closely linked to the pleasure of groove, involving activation of midbrain reward regions like the nucleus accumbens (Matthews et al., 2020). Moreover, rhythm is strongly associated with music’s emotional impact through changes in arousal, reflected in increased sympathetic nervous system activity (Khalfa et al., 2008; Rickard, 2004). Specific rhythmic properties, such as syncopation, positively impact emotional valence and enjoyment (Keller & Schubert, 2011). Our findings align with this body of evidence, offering a novel perspective on the link between rhythm and reward, where music reward sensitivity may also impact rhythm processing.

Research on the impact of affective stimuli on behavior supports our interpretation. Affective and reward-related cues influence brain information processing and behavior (Pessoa, 2009). Rewards enhance attention and executive functions, including working memory, cognitive flexibility, and response inhibition (Harlé et al., 2013; Pessoa, 2009) via reward-dependent dopamine release in the prefrontal cortex (Friedman & Robbins, 2022; Ott & Nieder, 2019). In the visual domain, mood manipulation improves the detection of emotional stimuli (Jolij & Meurs, 2011), and fearful faces increase contrast sensitivity (Phelps et al., 2006). Similarly, motivationally salient stimuli—whether emotional or economic—are perceived more readily than neutral ones (Bruner & Goodman, 1947; Veltkamp et al., 2008; Zadra & Clore, 2011).

In the music cognition domain, Trost and colleagues (2014) found that more pleasant (consonant) music, compared to less pleasant (dissonant) music, enhanced rhythmic processing and increased activity in the caudate nucleus, a region linked to both reward and rhythmic processing (Matthews et al., 2020). In the same vein, Lazzari and colleagues (2024) conducted a study where pairs of non-musicians performed an interpersonal tapping task, creating together either pleasant (consonant) or unpleasant (dissonant) chords. After each trial, participants rated the pleasure they experienced. The study revealed that greater subjective pleasure within the dyad predicted better interpersonal synchronization, with consonance having a stronger effect in dyads more sensitive to music reward. Individuals with higher musical hedonia experience greater pleasure from music, including its rhythmic component, through activation of reward and motor networks (Martinez-Molina et al., 2016; Matthews et al., 2020). In our study, the enhanced reward responses to rhythm and music in the Finger-Tapping and CA-BAT tasks may have influenced how participants processed rhythmic stimuli and allocated attention, affecting both rhythm perception and production performance.

Reward also influences how information is encoded in memory, with highly rewarding stimuli, such as monetary incentives or curiosity states, promoting information learning and consolidation through the hippocampal-mesolimbic dopaminergic pathway (e.g., Gruber et al., 2014; Lisman et al., 2011; Tsetsenis et al., 2023). In music research, studies have consistently shown that pleasurable musical stimuli are associated with better episodic memory performance, particularly in individuals with higher musical hedonia (Cardona et al., 2020; Ferreri & Rodriguez-Fornells, 2017, 2022; Ferreri et al., 2021). In this study, we did not find a significant relationship between reward processing and memory for rhythmic patterns, contrary to our initial hypothesis. This discrepancy may be due to the task’s focus on discrimination-recognition abilities rather than episodic processing, which might not have been sensitive enough to detect the influence of individual differences in music reward. However, additional analysis showed the expected significant relationship between musical hedonia and memory-based rhythm processing when looking for musical expertise and including only participants reporting 0 years of formal musical training. A possible explanation is that non-musicians rely even more heavily than musicians on the affective qualities of the stimulus when mnemonically processing rhythmic patterns. Musicians, on the other hand, might employ different strategies, possibly involving attentional or executive processes shaped by their musical training. This further underscores the importance of conducting a dedicated study specifically on musicians. In addition, model selection analysis of the eBMRQ subscales (on the total sample) revealed that the Sensory-Motor facet significantly predicted MET performance. This suggests that individuals who derive greater pleasure from sensory-motor responses to music may have enhanced rhythmic memory discrimination, consistent with established reward-memory links. Further supporting this, the Finger-Tapping task results showed that individuals who experience higher sensory-motor pleasure from music perform better in rhythmic production. This also aligns with recent evidence showing that the Sensory-Motor subscale of the eBMRQ is the strongest predictor of urge to move subjective ratings among all subscales, further highlighting its tight link with music-driven synchronization behavior (Benson et al., 2024). In contrast, the CA-BAT results suggested that other dimensions of music reward, such as Musical Seeking and Social Reward, may have a more significant impact on rhythm perception. For example, those who actively seek out music may, through greater and more varied musical exposure, develop a more finely tuned internal model, leading to enhanced rhythm perception processing. These findings underscore the need for future research to clarify how each subcomponent of music reward sensitivity might uniquely influence different aspects of rhythmic processing. It is worth noting that the correlational nature of the study does not allow for the exclusion of the opposite direction: greater rhythmic abilities might drive greater reward sensitivity to music. For example, a recent study on groove found that, in individuals with musical anhedonia (i.e., low or absent sensitivity to music reward), the urge to move mediates the link between rhythmic complexity and pleasure, suggesting that motor engagement may compensate for blunted pleasurable responses (Romkey et al., 2025). Accordingly, stronger rhythmic abilities might enhance the music pleasurable experience via its sensory-motor component. Nevertheless, and importantly, by showing that musical hedonia—and its distinct facets—are associated with rhythm perception, synchronization, and memory performance, our results emphasize the importance of an individual-differences approach in studying the rhythm-reward relationship.

Human sensitivity to rhythm and the ability to synchronize with music are evident from the earliest days of life and persist throughout daily activities (Winkler et al., 2009). However, there is considerable individual variability, with some individuals showing strong beat-synchronization abilities while others struggle to keep time (Grahn & Schuit, 2012; Mills et al., 2015). The interest in individual differences in rhythmic skills has driven research to explore the role of expertise. Studies in music research have shown that rhythm processing varies with musical training, with differences between musicians and non-musicians. In adults, highly trained musicians exhibit lower variability than non-musicians and amateurs in rhythmic tapping tasks (Repp & Su, 2013 for a review), with this effect being modulated by the type of trained instrument. For example, drummers showed less variability in synchronization tasks as compared to amateur pianists, singers, and non-musicians (Krause et al., 2010). Athletes—another category of movement-based experts—were found to be as precise as musicians in rhythmic tasks (a circle-drawing and a tapping task) compared to non-expert controls (Braun Janzen et al., 2014). Similarly, musicians outperform non-musicians in perception abilities, such as pitch discrimination (Micheyl et al., 2006; Spiech et al., 2023; Tervaniemi et al., 2005), and in auditory short-term memory (Talamini et al., 2017). In this context, our findings extend existing research by suggesting that, in individuals without musical expertise, rhythm-related skills are influenced by traits associated with reward processing, rather than sensorimotor training.

Disentangling the factors that drive rhythmic processing becomes crucial in clinical contexts, as rhythmic deficits are observed in Parkinson’s disease, ADHD, and dyslexia (e.g., Bégel et al., 2022; Flaugnacco et al., 2014; Puyjarinet et al., 2019), where rhythmic interventions have been shown to improve motor and language functions (Nave-Blodgett et al., 2021). Our findings suggest that music-induced reward responses and individual differences in musical hedonia may inform tailored rhythm-based therapies (Park, 2022; Grau-Sanchez et al., 2018).

This study indicates that higher musical hedonia, reflecting heightened reward circuit engagement, is closely linked to rhythmic production and perception. This underscores the complex interplay between rhythm and reward in musical experience (Fiveash et al., 2023). Given the correlational nature of our study, further experimental research is warranted to disentangle the effects of rhythm-driven pleasure on rhythmic performance, particularly in memory-based rhythm processing. Considering the potential role of musical expertise suggested by our data, it would also be interesting to investigate how musicianship may modulate the relationship between music reward and rhythmic processing, for example, by including and assessing a sample of expert musicians. Moreover, future studies could also manipulate stimulus pleasantness (e.g., varying rhythmic complexity, Witek et al., 2014) to directly assess its impact on rhythmic processing components.

In conclusion, our research highlights the connection between musical reward and rhythmic processing, including production and perception, opening avenues for future interdisciplinary studies to explore the applications of music’s rhythmic and rewarding properties in cognitive and clinical contexts.

## Supplementary Information

Below is the link to the electronic supplementary material.

Supplementary Material 1 (DOCX 429 KB)

## Data Availability

Data is provided upon request.
