# Supplementary material for: The relationship between individual sensitivity to music reward and rhythmic processing
Source: Psychol Res. 2026 Apr 9;90(2):70. doi: 10.1007/s00426-026-02276-8 (PMC13065602; doi:10.1007/s00426-026-02276-8)
Supplement: Supplementary file 1 — Supplementary Material 1 (DOCX 429 KB) [file 426_2026_2276_MOESM1_ESM.docx]

*Psychological Research Journal*

**The Relationship between Individual Sensitivity to Music Reward and Rhythmic Processing**

Eleonora Fullone^1-2^, Daniele Gatti^1^, Giorgio Lazzari^1^, Luca Rinaldi^1-3^,

Carlotta Lega^1^, Laura Ferreri^1^

^1^ Department of Brain and Behavioral Sciences, University of Pavia, Pavia, Italy

^2^ School for Advanced Studies - IUSS, Pavia, Italy

^3^ Applied Psychology Unit, IRCCS Mondino Foundation, Pavia, Italy

**Corresponding author:**

Eleonora Fullone, Department of Brain and Behavioral Sciences, University of Pavia, Piazza Botta 6, 27100 Pavia, Italy. *e-mail address:* eleonora.fullone01@universitadipavia.it

To further explore individual differences, we analyzed whether perceptual abilities (PA; Gold-MSI subscale) interact with musical hedonia (eBMRQ) in predicting task performance. The PA subscale was added to the main models for the Finger-Tapping, CA-BAT, and MET tasks, including its interaction with eBMRQ.

**Data analysis**

*Finger-Tapping task.* To investigate whether perceptual abilities influence the relationship between musical hedonia and participants’ tapping performance, we fitted a linear mixed-effects model (LMM) including the interaction between eBMRQ scores, Gold-MSI Perceptual Abilities scores (PA), and order of taps within each trial in the Continuation phase. Participants and ISI were included as random intercepts. *DeltaRTs* served as the dependent variable, while eBMRQ scores, PA scores, and order of taps were treated as continuous fixed factors, including all interactions.

*CA-BAT task.* To investigate whether perceptual abilities affect the relationship between musical hedonia and participants’ rhythmic perception, we estimated a GLMM with the proportion of asynchronous responses as the dependent variable. Fixed factors included BTAs, participants’ eBMRQ and PA scores, and all their interactions. Participants and tracks were included as random intercepts.

*MET task.* To examine whether perceptual abilities influence the relationship between musical hedonia and task performance, we estimated a linear model (LM) with participants’ d’ as the dependent variable. Continuous predictors included participants’ eBMRQ and PA scores, and their interaction.

**Results**

*Finger-Tapping task.* The Type III Analysis of Variance using Satterthwaite’s method showed no significant main effects of eBMRQ (F(1,126.0) = 0.29, p = .589) or PA (F(1,126.1) = 0.12, p = .728). The main effect of order of taps was significant (F(1,43379.1) = 6.18, p = .013), reflecting a general tendency toward slower responses as trials progressed. Significant interactions were observed between eBMRQ and order of taps (F(1,43379.1) = 5.98, p = .014) and between PA and order of taps (F(1,43379.1) = 12.17, p < .001). Crucially, the three-way interaction among eBMRQ, PA, and order of taps was also significant (F(1,43379.1) = 9.06, p = .003), suggesting that participants with higher perceptual abilities and greater sensitivity to music reward exhibited more stable tapping performance over time (**Fig. S1**). In conclusion, the main effect reported in the primary analysis remained robust and was maximized among participants with higher perceptual abilities.

*CA-BAT task.* The main effect of BTA was significant (χ²(1) = 1268.14, p < .001), reflecting a general tendency for participants to respond ‘asynchronous’ more often as the misalignment increased. The GLMM revealed no significant main effects of eBMRQ (χ²(1) = 1.36, p = .244) or PA (χ²(1) = 1.93, p = .165). Significant interactions were observed between BTA and PA (χ²(1) = 52.69, p < .001). Importantly, the three-way interaction among BTA, eBMRQ, and PA was also significant (χ²(1) = 3.85, p = .049), suggesting that participants with higher perceptual abilities and greater sensitivity to music reward showed the highest sensitivity to misalignment (**Fig. S2**). Thus, the effect reported in the primary analysis remained stable and was maximized among participants with higher perceptual abilities.

*MET task.* The model had an adjusted R² = .02 and revealed an F(3,115) = 1.87, p = .139, with the overall model not statistically significant. The main effects of individual differences in musical hedonia (eBMRQ; t = 0.16, b = 0.06, p = .872) and perceptual abilities (PA; t = 1.77, b = 0.01, p = .079) were not significant. Additionally, the interaction between eBMRQ *and* PA was also non-significant (t = -0.07, b = -0.00, p = .946). As shown in **Fig. S3**, these results indicate that neither musical hedonia nor perceptual abilities significantly influenced performance in this task, consistent with the main analysis.

**Discussion**

Overall, these results suggest that the impact of musical hedonia on Finger-Tapping and CA-BAT differs across individual differences in perceptual abilities, but not in the MET task. Specifically, the greater sensitivity to production and perception tasks in participants with higher musical hedonia was even stronger among those with higher perceptual abilities.

Although this analysis was exploratory and relied on a single self-assessed subscale, the finding nonetheless provides meaningful insight into how multiple types of individual differences—including perceptual abilities and sensitivity to musical hedonia—may jointly shape rhythmic processing. It also highlights the need for further research to investigate these effects in more detail and across a broader range of individual differences.

**
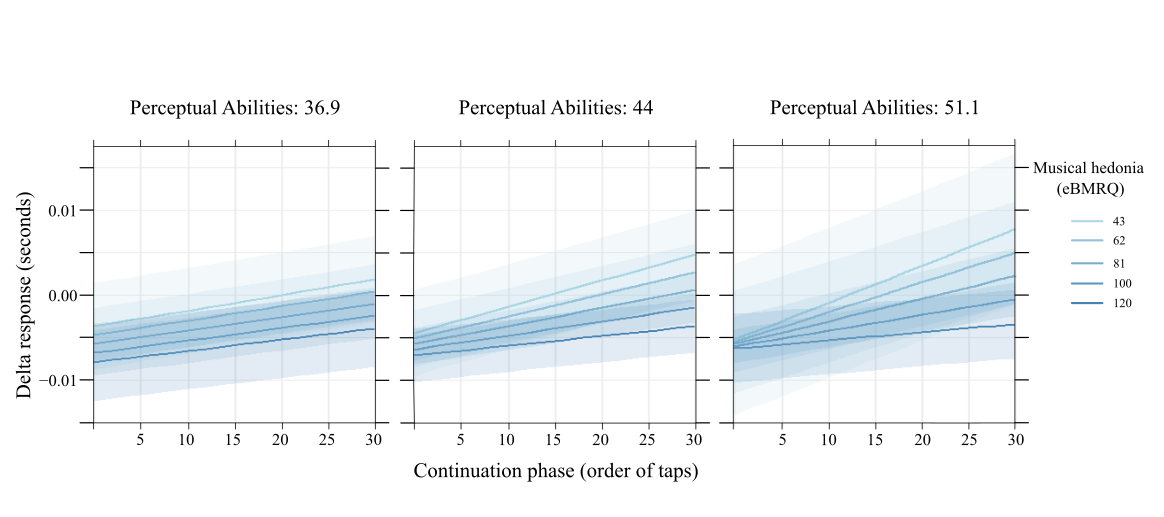
Figure S1.** Plots illustrating the results of the model estimated on Finger-Tapping data, illustrating the three-way interaction between musical hedonia (eBMRQ), perceptual abilities (PA), and the Continuation phase (order of taps). As shown, participants with higher musical hedonia exhibited more stable tapping over time (lower deltaRTs), indicating greater timing consistency, particularly when they also had higher perceptual abilities.


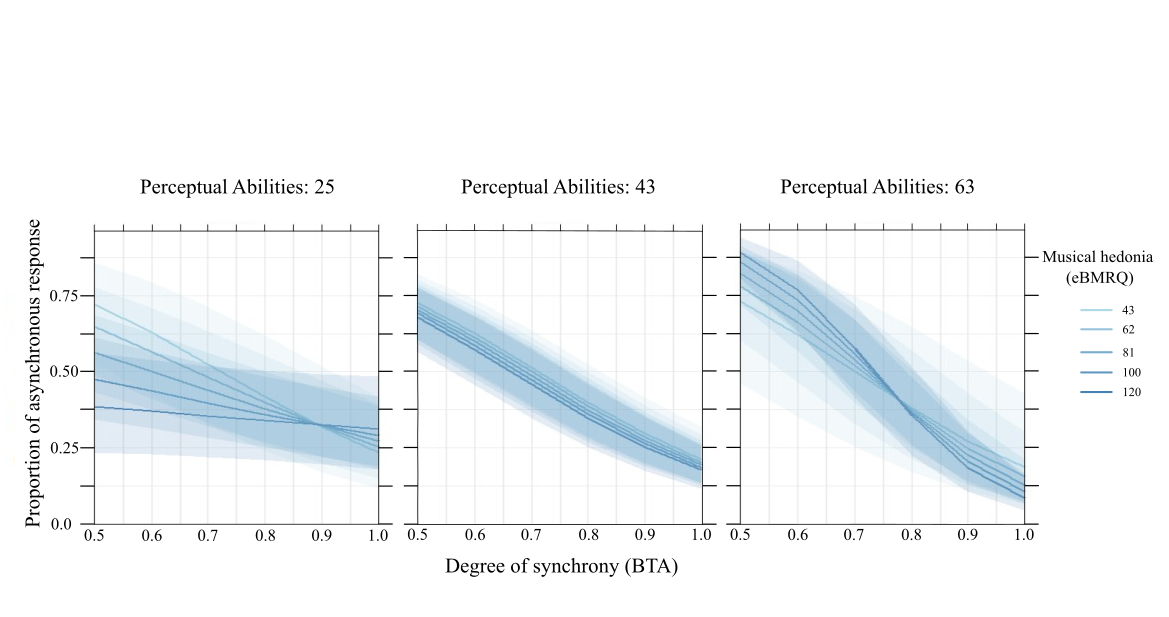


**Figure S2.** Plot illustrating the results of the model estimated on the CA-BAT task, showing the interaction between musical hedonia (eBMRQ), perceptual abilities (PA), and the degree of misalignment in the stimuli (BTAs). As shown, participants with higher musical hedonia exhibited greater sensitivity to misaligned stimuli (higher probability of responding ‘asynchronous’ for lower BTAs), indicating more precise rhythmic perception, but this effect was evident primarily in participants with higher perceptual abilities.


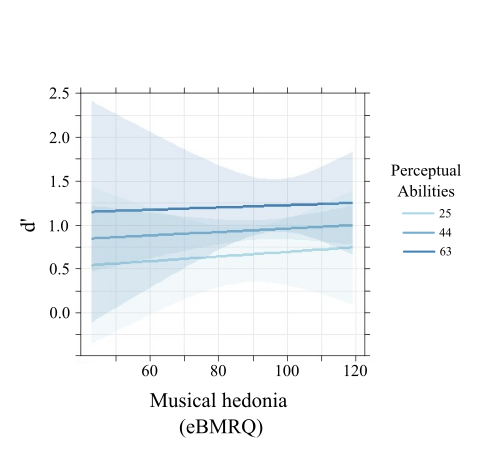


**Figure S3.** Plot illustrating the results of the model estimated on MET task data, showing the interaction between musical hedonia (eBMRQ) and perceptual abilities (PA) in predicting task performance (d’). As shown, neither musical hedonia nor perceptual abilities, nor their interaction, significantly predicted d’, indicating no modulatory effect of these individual differences on rhythmic memory.
